# Effective Decolorization of Poly-γ-Glutamic Acid Fermentation Broth by Integrated Activated Carbon Adsorption and Isoelectric Point Precipitation of Glutamic Acid

**DOI:** 10.3390/molecules29235769

**Published:** 2024-12-06

**Authors:** Xiusheng Si, Jicheng Li, Tingbin Zhao, Weifeng Cao, Changsheng Qiao

**Affiliations:** 1 College of Bioengineering, Tianjin University of Science and Technology, Tianjin 300457, China; sixiusheng@163.com (X.S.); 15665210180@163.com (J.L.); 2 Tianjin Engineering Research Center of Microbial Metabolism and Fermentation Process Control, College of Biotechnology, Tianjin University of Science and Technology, Tianjin 300457, China; 3 Tianjin Huizhi Biotrans Bioengineering Co., Ltd., Tianjin 300457, China; zhaotingbin369@163.com

**Keywords:** poly-γ-glutamic acid, adsorption, activated carbon, crystallization, integrated process

## Abstract

Poly-γ-glutamic acid (γ-PGA) is widely used in the field of biomedicine, food, agriculture, and ecological remediation. For the biosynthesis of γ-PGA, the pigments and remaining glutamate are two big problems that impede γ-PGA production by fermentation, and a trade-off between the decolorization rate and γ-PGA recovery rate during the purification process was found. The optimized static activated carbon adsorption conditions for treating the 2-times diluted cell-free supernatant (i.e., feed solution) was as follows: 0.51% 200-mesh, 1000 iodine value, coal-based activated carbon, pH 6.0, 140 min, and 40 °C. Under the optimized conditions, the decolorization rate reached 94.42%, and the recovery rate of γ-PGA was 94.22%. During the adsorption process, the pigments were adsorbed on the activated carbon surface in a monolayer, and the process was a spontaneous, heat-absorbing, and entropy-increasing process. Then, the decolorization flow rate optimized for the dynamic decolorization experiment was 1 BV/h. However, the remaining glutamate was still a problem after the activated carbon adsorption. After isoelectric point (IEP) precipitation of glutamic acid, the glutamic acid can be recovered, and the residual pigment can be further removed. Finally, an integrated decolorization process of activated carbon adsorption and IEP precipitation of glutamic acid was developed. After the integrated process, the decolorization and glutamic acid precipitation rates were 95.80% and 49.02%, respectively. The recovered glutamic acid can be reused in the next fermentation process.

## 1. Introduction

Poly-γ-glutamic acid (γ-PGA), linked by an amide bond between the monomer γ-carboxy and amino groups [1], is widely used in the field of biomedicine [2,3,4], food, agriculture [5], and ecological remediation [6]. *Bacillus* species have been reported to be the main genus for the biosynthesis of γ-PGA [7]. Depending on the substrate glutamic acid (Glu) for γ-PGA production, it was briefly classified into two categories: Glu-dependent fermentation and Glu-independent fermentation [8]. In Glu-dependent fermentation, exogenous L-Glu must be added to the broth for the strain to synthesis γ-PGA. For example, Guo et al. [8] obtained a high concentration of γ-PGA with the addition of 80 g/L monosodium glutamate, and the γ-PGA production decreased with the decreasing concentration of monosodium glutamate. Li et al. [9] also confirmed that a γ-PGA yield of 42.55 g/L with a productivity of 1.15 g/(L·h) was achieved in a medium supplemented with xanthate powder (FA powder, a glutamate substitute), whereas the γ-PGA yield was only 2.87 g/L in a medium without FA powder. Meanwhile, in Glu-independent fermentation, the strain can directly synthesize γ-PGA from the substrate in the broth without L-Glu. For example, Zeng et al. [10] obtained a yield of 19.50 g/L γ-PGA from the Glu-independent *B. subtilis* GXG-5. These results indicated that Glu-dependent fermentation can offer a much higher γ-PGA production than the Glu-independent fermentation case, while the exogenous L-Glu will also be a problem in the purification process.

Usually, γ-PGA-related standards have strict requirements on the color value of the samples, such as cosmetic grade γ-PGA being white and agricultural grade γ-PGA being off-white. In addition, pigment is the main impurity and color-generating substance in crude products, which needs to be removed in order to obtain a higher product appearance and meet the market demand. However, during the process, some technical problems, such as low ratios of decolorization and high γ-PGA loss, impede technological upgrading in the γ-PGA industry. Currently, a variety of decolorization methods have been reported, such as H_2_O_2_ oxidation, macroporous resin adsorption [11,12], and activated carbon adsorption [13]. H_2_O_2_ will hydrolyze to form HO_2_^−^ during decolorization, and its formation of HO_2_^−^ with strong oxidizing properties will oxidize pigments to achieve the purpose of decolorization [14]. However, the strong oxidizing property of H_2_O_2_ will destroy the structure of large molecular weight γ-PGA and is not suitable for the decolorization of γ-PGA fermentation broth. Macroporous resins have different types, and, in general, they can selectively adsorb target components from the system by electrostatic forces, hydrogen bonding interactions, and complexation [15], but the decolorization rate was low, which should be associated with other technology, such as ultrafiltration [11], for decolorizing the γ-PGA fermentation broth. Usually, activated carbon can simultaneously adsorb pigments and product. For example, using activated charcoal as a decoloring agent, only 85% protease could be recovered with a decolorization ratio of 90% during decolorizing the enzyme from a crude extract in solid state fermentation of wheat bran by *Rhizopus oryzae* [16]. However, activated carbon, which usually has the advantage of a higher specific surface area [17] and shorter time to reach adsorption equilibrium than macroporous resins, may be more suitable for the decolorization of γ-PGA fermentation broth. For the recovery of L-Glu, ultrafiltration (UF) was initially used to clarify the broth, and the L-Glu was recovered by isoelectric point (IEP) precipitation from crystalline and distilled UF permeate [18]. In addition, two electrodialysis processes, such as two-compartment bipolar membranes electrodialysis and modified traditional electrodialysis, were developed to recover L-Glu from IEP supernatant [19]. Thus, IEP precipitation was a universal method to recover L-Glu from its broth. However, in the γ-PGA broth system, the effects of residual L-Glu on the extraction process of γ-PGA, such as the decolorization rate and the recovery rate of γ-PGA, were still unknown. In addition, the effect of γ-PGA on the recovery rate of L-Glu by IEP precipitation needed to be evaluated.

In the study reported here, in order to fill the gap on the γ-PGA recovery rate, decolorization rate, and IEP precipitation of L-Glu, a process for the decolorization of γ-PGA fermentation broth with commercially available activated charcoal was evaluated, and the decolorization mechanism was also studied. Meanwhile, the effect of L-Glu on the decolorization was also analyzed. Finally, integrated activated carbon adsorption and isoelectric point precipitation of glutamic acid for the decolorization of γ-PGA was developed.

## 2. Results and Discussion

### 2.1. Decolorization by Activated Carbon

#### 2.1.1. Effect of Activated Carbon Particle Size on Decolorization Rate

**Figure 1:**
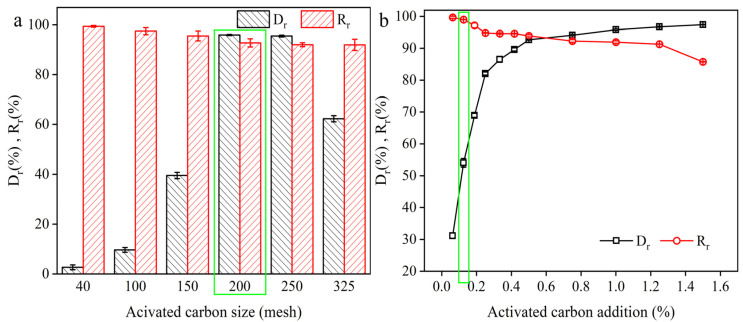
Effect of activated carbon sizes at the additive amount of 1% (**a**) and dosage using the 200-mesh, coal-based activated carbon (**b**) on the decolorization and recovery rates. The experiments were carried out at pH 7.0, 50 °C, and 50 min with different 1000 iodine value, coal-based activated carbons. The green frame shows the optimal parameter in the figure. Data are expressed as mean ± standard deviation (*n* = 3).

The adsorption function of activated carbon is determined by the loose microporous structure of the surface [20], and its adsorption characteristics are closely related to its particle size [21]. Thus, the effect of activated carbon particle size on the decolorization rate (D_r_, %) was first evaluated using a coal activated carbon with an iodine value of 1000 at pH 7.0, 50 °C, 50 min, and 1% dosage. As shown in Figure 1a, the decolorization rate increased with the decreasing particle size of the activated carbon when the particle size was below 200 mesh, and it was similar when the particle size was between 200 mesh and 250 mesh. However, the decolorization rate began to decrease when the particle size was above 250 mesh. Meanwhile, the γ-PGA recovery rate (R_r_, %) continued to decrease with the decreasing particle size of the activated carbon. For example, the decolorization rates were 2.66% with a γ-PGA recovery rate of 99.38% and 95.93% with a γ-PGA recovery rate of 92.76% at 40 mesh and 200 mesh, respectively. The phenomenon was in agreement with that reported by Seiki and Müller et al. [20,22,23], that is, activated carbons with small particle sizes showed good adsorption capacity. The reason may be that the specific surface area of the activated carbon increases as the particle size decreases, and activated carbon with small particle size possesses a larger specific surface area and therefore adsorbs a higher number of pigments, i.e., a higher decolorization rate. Surprisingly, the 325-mesh activated carbon was not as effective in its decolorization rate as the 200-mesh or 250-mesh, which was also supported by the finding of Matsui et al. [24]. They found better adsorption of 2-methylisoborneol (MIB) and geosmin on powdered activated carbon of medium particle size by using the branching pore kinetic model (BPKM) in conjunction with the Freundlich isotherm equation. In addition, since the market price of 250-mesh activated carbon is higher than that of 200-mesh activated carbon, the 200-mesh activated carbon was selected in the next sections.

#### 2.1.2. Effect of Activated Carbon Dosage on Decolorization Rate

**Figure 2:**
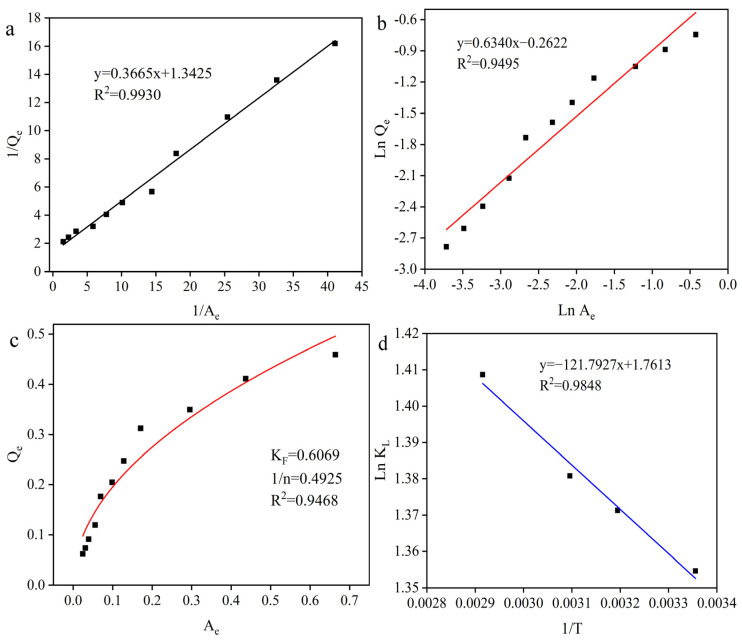
Linearized Langmuir isotherm (**a**), linearized Freundlich isotherm (**b**), Freundlich isotherm of pigment molecules on activated carbon (**c**), and Van’t Hoff plot of the Ln K_L_ of activated carbon adsorbed pigments relative to 1/T (**d**). The data in (**a**–**c**) came from Figure 1b, and the data in (**d**) came from Figure S1.

In order to further enhance the γ-PGA recovery rate, the effect of activated carbon dosage on the decolorization rate was evaluated with the 200-mesh activated carbon under 50 min, 50 °C, and pH 7.0 conditions. As shown in Figure 1b, the decolorization rate increased with the increasing dosage of the activated carbon. The decolorization rate had the largest slope between 0.0625% and 0.25% dosage (Figure 1b), representing the maximum decolorization efficiency at this stage. Then, when the activated carbon dosage exceeded 0.5%, the slope of the decolorization rate decreased significantly. Based on the above data, the adsorption capacity (Q_e_) of the activated carbon was calculated for fitting the Freundlich and Langmuir models [16,25,26,27] (Figure 2a,b). The Q_max_ and Langmuir constant K_L_ were calculated from the linear relationship between A_e_/Q_e_ and A_e_. K_F_ and n were calculated from the linear relationship between lnQ_e_ and lnA_e_. Comparing the correlation coefficients R^2^, both Langmuir and Freundlich equations described the experimental equilibrium adsorption data better, and the Langmuir equation fitted better than the Freundlich model over the range of concentrations studied. These results indicated that the pigments were adsorbed on the activated carbon surface in a monolayer [28]. After fitting the Freundlich model (Figure 2c), 1/n = 0.49, which suggested excellent adsorption performance of activated carbon [28]. Furthermore, after fitting the Langmuir isotherm model [29], the R_L_ = 0.17, which is in agreement with the above Freundlich fitting results [30]. However, the γ-PGA recovery rate continued to decrease with the increasing dosage of activated carbon. These results indicated that the activated carbon not only adsorbed the pigments in the feed solution but also the γ-PGA, which resulted in the decrease in the γ-PGA recovery rate. In addition, when the activated carbon dosage was 0.125%, the decolorization rate and γ-PGA recovery rate were 54.05% and 99.02%, respectively, which was also located in the largest slope of the decolorization rate range. Thus, in order to further enhance the γ-PGA recovery rate and simultaneously keep the high decolorization rate, the activated carbon dosage was fixed at 0.125% for studying the other influencing factors in the following sections.

#### 2.1.3. Effect of Activated Carbon Material on Decolorization Rate

**Figure 3:**
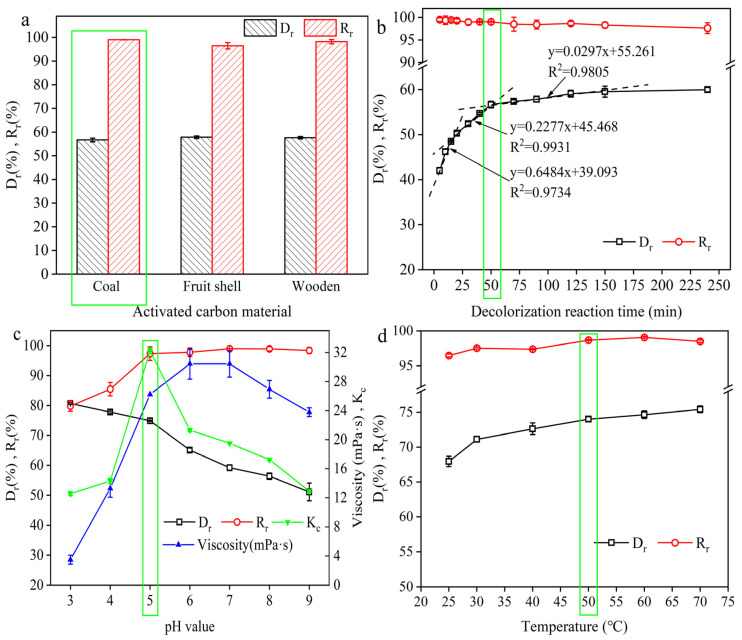
Effect of activated carbon material (**a**), adsorption time (**b**), pH (**c**), and temperature (**d**) on the decolorization and recovery rates. The experimental conditions were as follows: 1000 iodine value, 200-mesh coal activated carbon, 0.125% (*w*/*v*), 50 min, pH 7, and 50 °C. The green frame shows the optimal parameter in the figure. The data are expressed as mean ± standard deviation (*n* = 3).

As the physical and chemical properties of activated carbon determine its adsorption effect, the materials of activated carbon may affect its adsorption capacity. Thus, static decolorization experiments were carried out to investigate the differences in decolorization and recovery rate of feed solution for 200-mesh size, 1000 iodine value activated carbons made of different materials, including coal, wood, and fruit shells (Figure 3a), under 0.125% activated carbon addition (*w*/*v*), pH 7.0, 50 °C, and reaction time of 50 min conditions. As shown in Figure 3a, there was no significant difference between the above three types of activated carbon either in decolorization rate or γ-PGA recovery (*p* > 0.05), that is, the material of the activated carbon had no significant effect on the decolorization rate or γ-PGA recovery rate. Considering that the price of coal-based activated carbon was significantly lower than that of fruit shell activated carbon or wood-based activated carbon, coal-based activated carbon was selected. Moreover, to further investigate the effect of adsorption capacity of the coal-based activated carbon on the decolorization rate and the γ-PGA recovery rate of the feed solution, a 200-mesh, coal-based activated carbon with different adsorption parameters was used. Table 1 shows that the activated carbon with higher adsorption capacity has a higher decolorization rate, while the γ-PGA recovery rate showed a reverse trend. The reason was that activated carbon with high adsorption capacity contains a dense microporous structure, resulting in more adsorption structures under the condition of the same weight of activated carbon, so the adsorption capacity is stronger. Since the 1000 iodine value activated carbon showed a relatively high decolorization rate and low price, it was selected in the next sections.

#### 2.1.4. Effect of Decolorization Time on the Decolorization Rate

Decolorization time is another important factor affecting the decolorization rate [31], which was investigated under 50 °C, pH 7.0, and 0.125% activated carbon dosage with varied time conditions. As shown in Figure 3b, the decolorization rate increased with the decolorization time, and the γ-PGA recovery rate showed a reverse trend. For the decolorization rate, three lines were formed: y = 0.65x + 39.09 (R^2^ = 0.97) between 5 min and 15 min, y = 0.23x + 45.47 (R^2^ = 0.99) between 15 min and 50 min, and y = 0.03x + 55.26 (R^2^ = 0.98) between 50 min and 150 min. When the decolorization time was above 150 min, there was no significant effect of time on the decolorization rate. However, the γ-PGA recovery showed a decreasing trend with increasing decolorization time. The reason for this phenomenon may be that γ-PGA has reached the interior of the porous structure of the activated carbon due to prolonged contact, i.e., the adsorption of γ-PGA by the activated carbon increases after too long a decolorization time. Shao et al. [31] and Tao et al. [32] similarly reported the phenomenon that too long an adsorption time of the adsorbent being in the reaction system resulted in a serious loss of the target product, which is in agreement with the above findings. Meanwhile, it also clearly confirmed that the slope of the decolorization rate decreased significantly after 50 min, and the γ-PGA recovery rate reached 99.02%. Thus, considering work efficiency, 50 min was chosen for the subsequent experiments.

#### 2.1.5. Effect of pH on the Decolorization Rate

The degree of ionization of pigments is determined by pH, and pH also affects the van der Waals force between the pigments and the activated carbon. Thus, the effect of pH on the decolorization rate and γ-PGA recovery rate was explored under 50 °C, 50 min, and 0.125% activated carbon dosage with varied pH conditions (Figure 3c). For the decolorization rate, there was a significant increase with decreasing pH. One reason was that, when the pH value was below 6.0, the viscosity of the feed solution decreased with the decrease in pH value, which was favorable for the binding of pigment to the microporous structure of activated carbon. Another reason was that the residual glutamate has an isoelectric point (IEP) of 3.22. As the pH decreased, the ionization of glutamate decreased, and this affected the decolorization rate, which is evaluated in the next section. For γ-PGA recovery, the recovery of γ-PGA decreased when the pH was lower than 5.0, and the recovery of γ-PGA was extremely high when the pH was between 5.0 and 9.0 (i.e., above 97%). Then, the Zeta potential of the activated carbon was qualitatively detected in different pH environments, and the zero charge point of the used activated carbon was between pH 4 and pH 5. When the pH of the decolorization system was higher than 5, the activated carbon and γ-PGA were electronegative, and the adsorption effect between them was poor. However, there was a strong adsorption effect between the activated carbon and γ-PGA in other environments. Another reason was that the viscosity of the fermentation broth is low at lower pH, which was favorable for the combination of activated carbon and γ-PGA. In addition, since the IEP of γ-PGA is 3.47, the solubility of γ-PGA is lower under the conditions of pH 3 and pH 4, and the precipitation of crystals at the bottom of the feed solution can be observed, which can decrease the γ-PGA recovery rate. Moreover, Shi et al. [33] used the method of calculating the selection coefficients to determine the optimal one-factor conditions, which can ensure a high decolorization rate while taking into account the recovery rate. As shown in Figure 3c, K_c_ reached the maximum value at pH 5, which can preferably balance the decolorization rate and γ-PGA recovery rate. Thus, the pH 5 condition was chosen in the following sections.

#### 2.1.6. Effect of Temperature on the Decolorization Rate

Temperature is also a very important factor affecting the adsorption properties of activated carbon. Meanwhile, a higher temperature will reduce the viscosity of the feed solution and increase the movement speed of the pigments, which are favorable for the decolorization reaction. However, higher reaction temperatures will destroy the γ-PGA polymer structure and increase the power consumption, resulting in an increase in production costs. Therefore, in order to balance the decolorization rate and the γ-PGA recovery rate, the effect of temperature was studied under 50 min, pH 5.0, and 0.125% activated carbon dosage conditions (Figure 3d). As the temperature increased from 25 °C to 70 °C, the decolorization rate and γ-PGA recovery rate tended to increase with the increasing temperature. The reason for this phenomenon is that the viscosity of the feed solution decreases with increasing temperature as well as the intense Brownian motion of the pigments after the temperature increase, which is conducive to the combination of the pigments with the microporous structure in the activated carbon, which in turn exhibits a higher decolorization rate. In addition, the adsorption of pigments by activated carbon is not extremely strong, and the pigments can be desorbed from activated carbon by ethanol desorption. It is reasonable to assume that the adsorption of pigments by activated carbon is a kind of heat-absorbing reaction, and an increase in the temperature is favorable to the reaction for the heat-absorbing reaction. To test this hypothesis, we chose to fit the Langmuir model (Figure S1) and calculate the thermodynamic model (Figure 2d) at 25 °C, 40 °C, 50 °C, and 70 °C conditions. The thermodynamic parameters such as Gibbs free energy ΔG (kJ/mol), enthalpy change ΔH (kJ/mol), and entropy change ΔS kJ/(mol·K) in the experiments of decolorization of the feed solution by activated carbon are given in Table 2. The fitting results were as follows: ΔG was negative, indicating that the adsorption reaction was a spontaneous process at the experimental temperature. ΔH was positive, proving that the adsorption of pigments by the activated carbon was a heat-absorbing reaction, and that the adsorption was favored by an increase in temperature. ΔS was positive, indicating that the process was an entropy-increasing process [34].

#### 2.1.7. Response Surface Optimization of Decolorization Conditions

In order to further obtain optimal parameters for decolorization, four influencing factors and three levels of experimental conditions were designed using Design-expert 13 software to obtain 29 sets of RSM experiments [35,36]. The final experimental results are shown in Table S1. The experimental data were fitted to the following second-order polynomial equation model by regression analysis results [37]:

(1) $$\begin{array}{l} D_{r}\left(\%\right)=83.172+181.338A–11.432B+0.025C+0.241D+12.782\mathrm{AB}–0.060\mathrm{AC}–0.348\mathrm{AD}+\\ 0.003\mathrm{BC}+0.021\mathrm{BD}+0.0003\mathrm{CD}–237.642A^{2}+0.184B^{2}–0.00007C^{2}–0.003D^{2} \end{array}$$

where D_r_ is the decolorization rate (%); A, B, C, and D are the coded values of activated carbon dosage, feed solution pH, decolorization time, and decolorization temperature, respectively.

ANOVA, F-values, *p*-values, misfit, and R^2^ were used to assess the feasibility of the model and the interaction between variables. A model term was considered significant when the *p*-value was less than 0.05. In addition, a *p*-value > 0.05 for lack of fit indicates that it is not statistically significant relative to pure error. The significance of the fitted equations was tested by analysis of variance (ANOVA), as shown in Table S2. From the ANOVA results, its validity is indicated by its F-value and *p*-value of 446.06 and <0.0001, respectively. The probability of obtaining this “model F-value” due to error is less than 0.01%. In this case, A, B, C, D, AB, AC, AD, A^2^, and C^2^ were significant model terms (*p* < 0.05) while the rest of the terms were not significant (*p* > 0.05), suggesting that all four factors had a substantial effect on the decolorization rate of the feed solution. The degree of influence of these four factors on the decolorization rate of feed solution, judged by the F-value corresponding to each single factor, was in the following order: activated carbon addition > feed solution pH > decolorization time > decolorization temperature. As shown in Table S3, the results showed that the R^2^ and the adjusted R^2^(Adj) were 0.9978 and 0.9955, respectively. The value of R^2^ describes the degree of estimation of the model on the experimental data points, and the adjusted R^2^ measures the change in the mean value of the model. Therefore, the model has high significance. The R^2^(Pred) value was 0.9886, with a difference from the R^2^ value of < 0.2. The results indicated that the experimental data on the decolorization rate of the feed solution were in good agreement with the predictions of the model. The coefficient of variation (C.V. %), which is a measure of relative variability, showed a C.V. % of 0.9230, with lower C.V. % indicating that the data points were around the mean.

**Figure 4:**
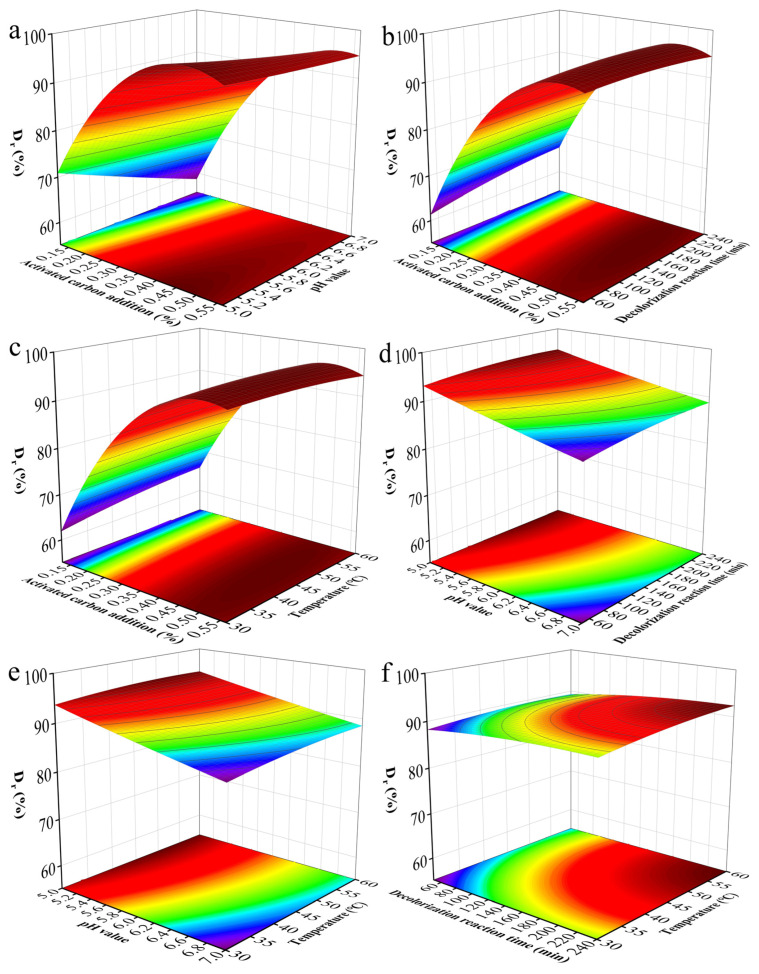
Response surface plots for the decolorization rate of γ-PGA showing the interactions between activated carbon dosage and pH (**a**), activated carbon dosage and decolorization time (**b**), activated carbon dosage and temperature (**c**), pH and decolorization time (**d**), pH and temperature (**e**), and decolorization time and temperature (**f**).

RSM was used to further analyze the four factors mentioned above in influencing the interaction and optimal level of the decolorization rate of the feed solution. Figure 4 shows the 3D response surface curves and the corresponding contour plots. According to the results of the response surface method, when the activated carbon addition (*w*/*v*) was 0.512%, the pH value of the feed solution was 6.08, the decolorization time was 139.4 min, and the decolorization temperature was 39.7 °C, the maximum predicted decolorization rate of the feed solution was 96.08%.

In summary, for the convenience of the subsequent experiments, the activated carbon addition (*w*/*v*) was taken as 0.51%, pH 6.0, decolorization time as 140 min, and decolorization temperature as 40 °C to verify the reliability of the model, and the validation experiments were carried out under the above experimental conditions, with a total of three parallel experiments. The results of the validation experiments were 94.39%, 94.29%, and 94.57%, respectively. It can be concluded that the average decolorization rate obtained from the three repeated experiments is 94.42%, which is basically consistent with the predicted value of 96.08%. It can be seen that the relationship model of activated carbon addition, pH value of feed solution, decolorization time, decolorization temperature, and decolorization rate of the feed solution designed and established by Design-expert software is accurate and reliable.

#### 2.1.8. Effect of Flow Rate on Decolorization Rate

**Figure 5:**
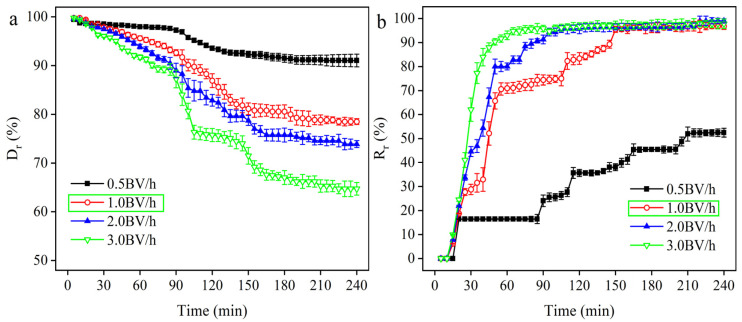
Effect of dynamic experimental flow rate on decolorization rate (**a**) and recovery rate (**b**). The experiments were carried out at pH 6.0 and 40 °C. The green frame shows the optimal parameter in the figure. The data are expressed as mean ± standard deviation (*n* = 3).

According to the optimal decolorization parameters obtained from the response surface mentioned above, dynamic decolorization experiments were carried out on the HXK column at a feed solution temperature of 40 °C and a pH value of 6.0 to investigate the dynamic leakage curves of activated carbon at different flow rates. The dynamic curves are shown in Figure 5a,b. With the extension of dynamic decolorization time, the recovery of γ-PGA increased, while the decolorization rate showed a decreasing trend, and, finally, the two were close to equilibrium, which was due to the limited adsorption capacity of a certain mass of activated carbon. With the increase in feed volume, the adsorption capacity of the activated carbon gradually tends to saturation, resulting in a gradual decrease in the decolorization rate. At the same time, the outflow of γ-PGA from the activated carbon column increased, and, thus, the recovery rate increased. Finally, when the activated carbon column reached adsorption equilibrium, the decolorization rate and γ-PGA recovery were close to equilibrium [32]. The highest decolorization rate at 0.5 BV/h was attributed to the long contact time between the feed solution and the activated carbon column at low flow rates, and, therefore, the best adsorption performance was achieved at the lowest flow rate of 0.5 BV/h. The reason may be due to the long contact time between the pigments and the activated carbon column, where the pigments interacted with the active microporous sites, leading to the full adsorption of the pigments in the activated carbon column. The effect of flow rate on decolorization efficiency is bidirectional [38]. As the flow rate increases, the decolorization rate decreases and the γ-PGA recovery increases, which is attributed to the reduction of pigments adsorbed on the activated carbon column by passing through the activated carbon column at a higher flow rate. Considering the adsorption capacity of the activated carbon column, the lower the flow rate, the longer the decolorization time and adsorption cycle [39]; therefore, 1.0 BV/h was selected as the optimal flow rate.

### 2.2. Decolorization by Isoelectric Point Precipitation of Glutamic Acid

**Figure 6:**
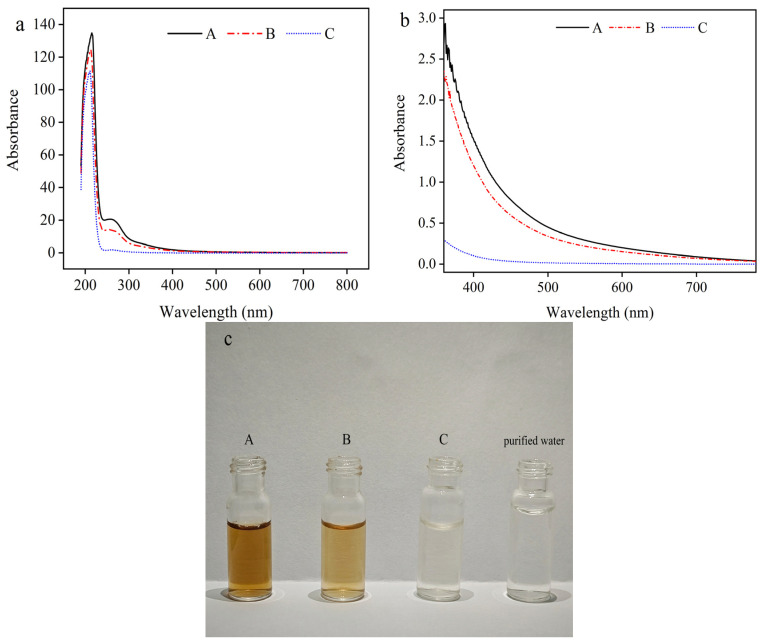
The UV–Vis spectra (**a**), Vis spectra (**b**), and photograph (**c**) of solutions before treatment (i.e., feed solution) (A), after IEP precipitation treatment (B), after the integrated treatment (C), and the control purified water. The experimental conditions of sole activated carbon decolorization were 0.51% activated carbon, 140 min, pH 6.0, and 40 °C.

At the end of the fermentation, the remain monosodium glutamate in the feed solution was 68 g/L, which may affect the decolorization rate by activated carbon. Thus, L-Glu was planned to be removed before decolorization by activated carbon. Surprisingly, it was found that the pigments in the feed solution decreased by 31.65% during the removal of L-Glu by IEP precipitation. The reason for this phenomenon may be that L-Glu carries part of the pigment along with it during the precipitation process due to electrostatic force. To confirm this hypothesis, the pigments adsorbed on activated carbon were desorbed by 60% ethyl alcohol, and the pigments was redissolved in deionized water after the ethyl alcohol was removed. It was found that the pH of the solution was below 7.0, which indicated that the pigment was acidic. Thus, during the precipitation of L-Glu, some pigments can be removed with the L-Glu, which can be clearly detected in Figure 6. In addition, the recovered L-Glu can be reused in the fermentation process. Thus, the recycling of monosodium glutamate in the feed solution can not only reduce the production cost but also reduce the generation and emission of waste, which is conducive to environmental protection and ecological balance.

### 2.3. Decolorization by the Integrated Activated Carbon Adsorption and IEP Precipitation of Glutamic Acid

**Figure 7:**
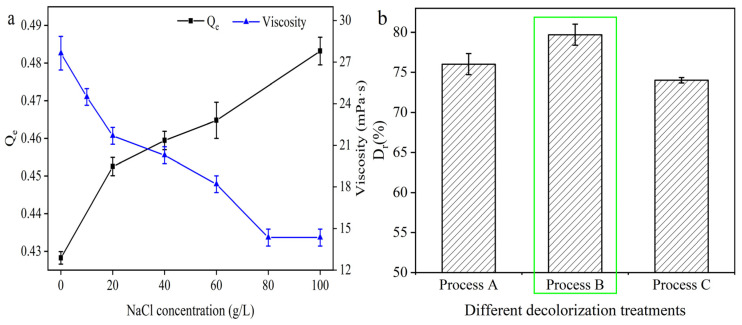
Effect of ionic strength (**a**) and decolorization process (**b**) on the decolorization rate. The experimental conditions were as follows: 0.125% activated carbon, 50 min, pH 5, and 50 °C. The green frame shows the optimal parameter in the figure. The data are expressed as mean ± standard deviation (*n* = 3).

After IEP precipitation of L-Glu, the remain L-Glu was decreased. As a result, the ionic strength in the feed solution decreases prior to activated carbon adsorption. Moreover, it has been confirmed that ionic strength showed a strong effect on the adsorption reaction [29]. The reason was that salt ions can compete with solutes for adsorption or affect the dissociation equilibrium of solutes [40]. For the adsorption of pigments on activated carbon, salt ions may diffuse into the microporous structure of the activated carbon and compete with the pigments for adsorption sites on the activated carbon and may also affect the degree of ionization of the pigments and interfere with the activated carbon adsorption effect. Thus, to evaluate the effect of ionic strength on the decolorization rate, different concentrations of NaCl were added in the feed solution, and the decolorization tests were carried out under 50 min, pH 5.0, and 0.125% activated carbon dosage conditions. Surprisingly, the adsorption of pigment by activated carbon (Q_e_) increased with increasing NaCl concentration, as shown in Figure 7a, which is inconsistent with the findings of Li et al. [40]. Furthermore, it was found that the viscosity of the feed solution was significantly reduced after the addition of NaCl, which increased in the contact area between the activated carbon and the feed solution, thus improving the decolorization rate.

Based on these results, the sequence of the IEP precipitation steps of L-Glu and the activated carbon adsorption was explored, and the decolorization tests were carried out under the best conditions optimized from the one-way experiments described above (i.e., 50 min, pH 5.0, and 0.125% activated carbon dosage). The process of decolorization was defined as follows: Process A (i.e., IEP precipitation of glutamic acid + activated carbon adsorption), Process B (i.e., activated carbon adsorption + IEP precipitation of glutamic acid), and Process C (i.e., activated carbon adsorption). As shown in Figure 7b, the decolorization rates of the three processes were in this order: Process B > Process A > Process C. Meanwhile, it was found that the viscosity of the feed solution after the IEP precipitation of L-Glu was increased from the original 27.64 mPa·s to 30.44 mPa·s, which would lead to the difficulty of the pigments to combine with the microporous structure of the activated carbon. These results were consistent with the phenomenon observed by the addition of NaCl. After three parallel experiments using process B, the precipitation rate D_g_ of L-Glu was 49.02 ± 1.70%, and these wet glutamates could be reused in the γ-PGA fermentation process after re-solubilization.

Moreover, the adsorption mode of activated carbon is non-selective adsorption [41], i.e., obtaining a higher decolorization rate of feed solution will result in a greater loss rate of γ-PGA. In order to solve the above problems, the use of an integrated decolorization process (i.e., Process B) can reduce the amount of activated carbon and ensure the maximum γ-PGA recovery rate. The specific effects are shown in Table 3. It can be seen that the decolorization rate of the feed solution of the integrated process under the condition of activated carbon addition of 0.45% (*w*/*v*) is still the same as the decolorization effect of the activated carbon adsorption process alone when the activated carbon addition of 0.51% (*w*/*v*) was used. After the feed solution was treated with the integrated process, the color was similar to that of purified water (Figure 6).

## 3. Materials and Methods

### 3.1. Chemicals and Materials

Analytically pure NaOH and HCl were purchased from Komeo Chemical Reagent Co., Ltd. (Tianjin, China). γ-PGA fermentation broth was provided by Tianjin Beiyang Baichuan Biotechnology Co., Ltd. (Tianjin, China). The activated carbon was purchased from Henan Zhongju Purification Materials Co., Ltd. (Zhengzhou, China). The food-grade diatomaceous earth filter aid was purchased from Linjiang Shengmai Diatomaceous Earth Functional Material Company Limited (Baishan, China).

### 3.2. Pretreatment of Activated Carbon and Preparation of the Feed Solution

For activated carbon pretreatment, each type of activated carbon before use was pretreated in turn as follows: treated with 2.5% (*w*/*v*) NaOH solution for 4 h, washed with deionized water until neutral, treated with 5% (*v*/*v*) HCl solution for 4 h, washed with deionized water until neutral, dried at 80 °C to a constant weight, and sealed at 25 °C. For preparation of cell-free broth containing γ-PGA, the γ-PGA was biosynthesized by *B. licheniformis* BYBC1.21056, and the specific composition of the medium and the fermentation conditions are shown in the Supplementary Data. At the end of the fermentation, an equal volume of deionized water was added to the broth, and then the cells of *B. licheniformis* BYBC1.21056 were removed from the broth in time, and the resulting supernatant (i.e., feed solution) was immediately stored at −18 °C.

### 3.3. Decolorization Using Activated Carbon

#### 3.3.1. Static Decolorization Experiment

The static decolorization experiment was performed as follows: 30 mL of feed solution was added to a 50 mL centrifuge tube and then preheated to a set temperature; activated carbon was weighed using a TP-214 electronic analytical balance (Beijing Sartorius Instrument Systems Co., Ltd., Beijing, China) and added to the same centrifuge tube; the mixture was shaken at a constant temperature for some time, and, after the reaction, the activated carbon was removed using a 0.22 μm microporous filter membrane. By comparing the decolorization rate of the feed solution and the recovery rate of γ-PGA, the optimal type of activated carbon for decolorization was selected. Moreover, in order to further optimize the process parameters, four factors and 29 experiments, designed by Box–Behnken Design (BBD) [42,43], were conducted with five repetitions at the center point (Table 4).

#### 3.3.2. Dynamic Decolorization Experiment

Firstly, the selected decolorized activated carbon and filter aid were mixed well and then filled into a HXK26800 chromatography column (Beijing RuiDa HengHui Science and Technology Development Co., Ltd., Beijing, China) with the appropriate height. Secondly, the column was washed with five times of the activated carbon column volume (BV) with deionized water. Finally, the feed solution was continuously loaded onto the chromatographic column at a constant flow rate, and the samples were collected every 5 min. The feed solution flow rate was optimized by comparing the decolorization rate and γ-PGA recovery rate.

### 3.4. Decolorization Using Isoelectric Point Precipitation of Glutamic Acid

The IEP precipitation step was accomplished by adding α-glutamic acid crystals to the feed solution, slowly adding acid, and stepping down the temperature with continuous stirring until the pH was adjusted to 3.22, and the temperature was maintained at 4 °C. The specific method for IEP precipitation is described in the Supplementary Data.

### 3.5. Analytical Methods

The absorbances of the feed solution before and after the activated carbon adsorption [44] were determined at 440 nm using a PV3 UV spectrophotometer (Shanghai Mepda Instrument Co., Ltd., Shanghai, China), and the decolorization rate was calculated according to Shi et al. [33] (see Supplementary Data). The method for detection of γ-PGA was the same as described in Guo et al. [8] (see Supplementary Data). When exploring the effect of pH on the adsorption performance of activated carbon, a selectivity coefficient was introduced to facilitate the quantitative calculation. The determination of A_D0_, A_D1_, C_R0_, and C_R1_, the partition coefficients (K_d_), and the selection coefficients (K_c_) were calculated according to the method of Shi et al. [33] (see Supplementary Data).

Both the Freundlich and the Langmuir models were used to fit the decolorization data [16,28,45,46]. Moreover, in order to further study the thermodynamic properties of activated carbon decolorization [29,47] and the thermodynamic parameters of the adsorption process at different temperatures, pH 5.0, and decolorization time of 50 min, the relevant thermodynamic equations and explanations are given in the Supplementary Data.

The L-Glu precipitation rate was expressed by the following formula, and the concentration of L-Glu was detected using an SBA-40E (Institute of Biology, Shandong Academy of Sciences, Jinan, China) biosensor equipped with an L-Glu oxidase electrode [44]:

(2) $$D_{g}\left(\%\right)=\frac{C_{g0}-C_{g1}}{C_{g0}}\times 100\%$$

where C_g0_ and C_g1_ are the concentrations of L-Glu detected by the SBA-40E biosensor before and after L-Glu deposition, respectively. D_g_% is the L-Glu deposition rate.

### 3.6. Characterization by pH at the Zero Charge Point of Activated Carbon

To a 50 mL centrifuge tube, 0.102 g of activated carbon and 20 mL of pure water were added, and the pH of the system was adjusted to 1–13 using 6 mol/L HCl solution or 6 mol/L NaOH solution, respectively, and vortexed and oscillated and then left to stand for 24 h. The diluted activated carbon suspension was qualitatively analyzed for Zeta potential using a nanoparticle size and Zeta potential analyzer model BeNano 90 Zeta (Dandong Bettersize Instruments Ltd., Dandong, China).

## 4. Conclusions

This study demonstrated the feasibility of the integrated decolorization process of activated carbon adsorption and IEP of glutamic acid for the decolorization of γ-PGA feed solution from glutamate-dependent fermentation. In the first stage, the activated carbon adsorption process removed most of the pigments in the feed solution, and the recovery rate of γ-PGA was as high as 94.22%. In the second stage, IEP was used to further decolorize the feed solution and recover L-Glu, and the pigment, adsorbed by L-Glu, could be further removed. Moreover, the recovered glutamic acid could be recycled in the fermentation process. After the above two steps, the decolorization rate and glutamate precipitation rate of the feed solution were 95.80% and 49.02%, respectively. Furthermore, the decolorization strategy developed in this study was applicable to not only the Glu-dependent fermentation but also the Glu-independent fermentation where the IEP of glutamic acid process was not needed.

## Figures and Tables

**Table 1:** Effects of different coal activated carbons on the decolorization and recovery rates ^a^.

Adsorption Parameters	D_r_ (%) ^b^	R_r_ (%) ^b^	Price (US Dollar/Ton)
800 iodine value	41.64 ± 1.48	99.28 ± 0.52	814
1000 iodine value	53.84 ± 1.38	99.02 ± 0.06	1500
14 mL/0.1 g methylene blue value	45.85 ± 0.66	97.64 ± 1.59	1642
18 mL/0.1 g methylene blue value	62.15 ± 0.53	96.79 ± 0.86	1928
19 mL/0.1 g methylene blue value	73.61 ± 0.75	95.48 ± 0.88	2642
20 mL/0.1 g methylene blue value	77.08 ± 0.32	93.74 ± 0.95	3285

^a^ The experiments were carried out at pH 7.0, 0.125% (*w*/*v*) activated carbon, 50 °C, and 50 min. ^b^ The decolorization and recovery rates of PGA are expressed as mean ± standard deviation (*n* = 3).

**Table 2:** Thermodynamic parameters of adsorption reaction ^a^.

T (K)	K_L_	ΔG (KJ/mol)	ΔH (KJ/mol)	ΔS (J/mol·K)	R^2^
298	3.88	−3.35	−1.01	14.64	0.98
313	3.94	−3.56			
323	3.98	−3.70			
343	4.09	−4.01			

^a^ The experiments were carried out at pH 5.0 and 50 min.

**Table 3:** Comparison of the decolorization rates with different activated carbon dosages and decolorization processes.

Activated Carbon Addition (*w*/*v*)	D_r_ (%) from Sole Activated Carbon Adsorption	D_r_ (%) from Integrated Process
0	0	31.65 ± 1.01 ^a^
0.45%	91.55 ± 0.95	93.66 ± 1.44
0.48%	92.71 ± 1.33	94.60 ± 0.64
0.51%	94.40 ± 0.12	95.80 ± 0.98
0.54%	95.34 ± 0.53	96.25 ± 0.44

^a^ Data are expressed as mean ± standard deviation (*n* = 3).

**Table 4:** Levels of factors used in Box–Behnken experimental design.

Variable	Label	Level		
		−1	0	1
A	Activated carbon addition (%)	0.125	0.34375	0.5625
B	pH value	5.0	6.0	7.0
C	Decolorization time (min)	50	145	240
D	Decolorization temperature (°C)	30	45	60

## Data Availability

Data is contained within the article or Supplementary Material.

## Supplementary Materials

The following supporting information can be downloaded at: https://www.mdpi.com/article/10.3390/molecules29235769/s1, Table S1. Experimental protocols and response values obtained for the Box–Behnken design. Table S2. Analysis of variance of decolorization rate according to response surface quadratic model. Table S3. Model summary statistics. Figure S1. Langmuir model fitting at different temperature conditions, 25 °C (a), 40 °C (b), 50 °C (c), and 70 °C (d). The experiments were carried out at pH 5.0 with a decolorization time of 50 min.
