# Supplementary material for: Effective Decolorization of Poly-γ-Glutamic Acid Fermentation Broth by Integrated Activated Carbon Adsorption and Isoelectric Point Precipitation of Glutamic Acid
Source: Molecules. 2024 Dec 6;29(23):5769. doi: 10.3390/molecules29235769 (PMC11643718; doi:10.3390/molecules29235769)
Supplement: Supplementary file 1 [file molecules-29-05769-s001.zip › molecules-3327521-supplementary.pdf]

Supplementary Data

Effective decolorization of poly-γ-glutamic acid fermentation broth by integrated activated carbon adsorption and isoelectric point precipitation of glutamic acid

Xiusheng Si[a,b,c], Jicheng Li[a,b,c], Tingbin Zhao, Weifeng Cao*[,a], Changsheng Qiao*[,a,b,c]

[a]*College of Bioengineering, Tianjin University of Science and Technology, Tianjin 300457, China*
[b]*Tianjin Engineering Research Center of Microbial Metabolism and Fermentation Process Control, College of Biotechnology, Tianjin University of Science and Technology, Tianjin 300457, China*
[c]*Tianjin Huizhi Biotrans Bioengineering Co., Ltd, Tianjin 300457, China*

* Corresponding author:
Weifeng Cao. E-mail: weifcao@126.com.
Changsheng Qiao. E-mail: qiaochangsheng@163.com
Address: College of Biotechnology, Tianjin University of Science and Technology, Tianjin 300457, PR China

Fermentation conditions covered in Materials and Methods

Composition of the medium and fermentation parameters

Medium components: LB medium: NaCl 10g/L, tryptone 10g/L, yeast leaching powder 5g/L, agar powder 20g/L. Seed medium: glucose 30g/L, yeast paste 7g/L, tryptone 10g/L, magnesium sulfate heptahydrate 0.5g/L, dipotassium hydrogen phosphate 0.5g/L. Fermentation medium: monosodium glutamate 102.6g/L, glucose 18.3g/L, corn syrup 15.2g/L, ferrous sulfate 0.7g/L. The initial pH of LB medium and seed medium should be adjusted to 7.2 with NaOH solution, and the initial pH of fermentation medium should be adjusted to 6.8. The pH is not adjusted during seed culture and fermentation culture.

Fermentation conditions: The strain inoculated in LB medium was transferred to seed medium and incubated as a primary seed solution for 12 hours at 37 °C with shaking. The primary seed solution was transferred to the secondary seed fermenter for cultivation with a secondary seed medium volume of 15 L, an inoculum volume of 0.25%, an incubation temperature of 37 °C, a rotational speed of 300 rpm, an aeration volume of 2.2 vvm, a tank pressure of 0.03 MPa, and an incubation for 14 hours to obtain the secondary seed solution. The secondary seed solution was introduced into the fermenter at 10% inoculum. The fermentation temperature was 37 °C; rotational speed was 300 rpm;, aeration volume was 0.6 vvm; tank pressure was 0.03 MPa; and the fermentation was carried out for 72 hours to obtain γ-PGA fermentation broth.

Operations and testing methods covered in the article

Detailed steps for L-glu isoelectric point (IEP) precipitation

The decolorized fermentation broth was controlled at 25 °C and pH 5.0, and the temperature was slowly lowered, and acid was added, to wait for the precipitation of glutamic acid crystal nuclei. After the appearance of glutamic acid nuclei, 0.3% (w/v) of α-glutamic acid crystal seed was put into the fermentation broth, and the addition of acid and temperature reduction were stopped, and the broth was stirred for 2 hours. Then, we continued to slowly add acid and slowly reduce the temperature; 4 hours to reduce the pH of the fermentation broth to 3.22; then stopped adding acid and continued to reduce the temperature to 4 °C; then, continuous stirring and crystal maintenance for 10 hours; and, finally, natural precipitation for 4 hours; centrifugation could obtain wet glutamic acid.

Detection of γ-PGA concentration

γ-PGA concentration was determined as follows: The concentration of γ-PGA was analyzed using a high-performance liquid chromatography (HPLC) analyzer (Shimadzu, Japan). The mobile phase was 0.05 M Na_2SO_4 solution (chromatographic purity Tianjin Winda Rare Chemical Reagent Factory), flow rate: 0.5 mL/min, and the column was a SB-806 HQ gel permeation chromatographic column (Shodex, Japan), injection volume: 20 μL.

The mathematical equations and meanings involved in the discussion

Calculation of decolorization rate

The decolorization rate of γ-PGA fermentation broth by activated carbon was expressed by the following equation:

$$D_r(\%)=\frac{A_{D0}-A_{D1}}{A_{D0}}\times100\% \quad (1)$$

where A_{D0} and A_{D1} are the absorbance at 440 nm of the sample solution before and after decolorization of the fermentation broth, respectively. D_r% is the decolorization rate of γ-PGA fermentation broth.

Calculation of recovery rate

The following formula was used to calculate the recovery of γ-PGA:

$$R_r(\%)=\frac{C_{R1}}{C_{R0}}\times 100\% \quad (2)$$

where C_{R0} and C_{R1} are the concentrations of γ-PGA detected by HPLC in the samples before and after decolorization of γ-PGA fermentation broth, respectively. R_r% is the recovery of γ-PGA.

Adsorption isotherm fitting for activated carbon adsorption reactions

The degree of fit to Freundlich and Langmuir equations was evaluated. For adsorption of solutes in dilute solutions, the Freundlich equation is expressed as follows:

$$Q_e=\frac{V(A_0\text{-}A_e)}{M} \quad (3)$$

$$Q_e=K_F\times A_e^{1/n} \quad (4)$$

where Q_e is the apparent adsorption capacity per unit mass of adsorbent; V is the volume of fermentation broth (mL); M is the mass of activated carbon (g); K_F and n are constants; and the value of 1/n is an indicator of adsorption characteristics.

The Langmuir equation is expressed as follows:

$$Q_e=\frac{Q_{MAX}K_LA_e}{1+K_LA_e} \quad (5)$$

where A_e is the equilibrium concentration of free solute in solution, and Q_{max} and K_L are constants that depend on the adsorbent and the adsorbed substance's own properties. The plot of $1/A_e$ versus $1/Q_e$ will be a straight line.

The basic features of the Langmuir isotherm can be expressed in terms of a dimensionless constant called the separation factor R_L.

$$R_L=\frac{1}{1+K_L C_0} \tag{6}$$

where K_L is Langmuir's constant, and C_0 is the initial concentration of the adsorbed solute.

Mathematical equations for selection coefficients related to pH value selection in one-way experiments

$$K_{d1}=\frac{(A_{D0}-A_{D1})/M}{A_{D1}} \tag{7}$$

$$K_{d2}=\frac{(C_{R0}-C_{R1})/M}{A_{R1}} \tag{8}$$

$$K_c=\frac{K_{d1}}{K_{d2}} \tag{9}$$

where K_{d1} and K_{d2} are the decolorization constant and γ-PGA loss constant of the fermentation broth; A_{D0} and A_{D1} are the pigment absorbance before and after decolorization; C_{R0} and C_{R1} are the concentration of γ-PGA before and after decolorization, respectively; M is the mass of activated carbon used; and K_c is the coefficient of selective decolorization.

Thermodynamic fitting of the adsorption reaction to activated carbon

This was explored including Gibbs free energy change (ΔG), enthalpy change (ΔH) and entropy change (ΔS). ΔG can be calculated by the following equation:

$$\Delta G=-RT\ln K_L \tag{10}$$

where K_L is Langmuir's constant; T is the thermodynamic temperature in K; and R is the gas constant 8.314×10^{-3}, KJ/(mol·K).

The relationship between K_L and ΔH and ΔS can be described by the Van't Hoff formula:

$$\ln K_L = \frac{\Delta S}{R} - \frac{\Delta H}{RT} \qquad (11)$$

ΔH and ΔS can be calculated by plotting the slope and intercept of 1/T against $\ln K_L$.

Table S1

Experimental protocols and response values obtained for the Box–Behnken design.

Run	Coded variable level				Real variable level				D_r(%)
	A	B	C	D	Activated Carbon Addition (%)	pH value	Time (min)	Temperature (°C)	
1	0	0	0	0	0.34375	6	145	45	90.706
2	1	0	-1	0	0.5625	6	50	45	94.203
3	1	0	1	0	0.5625	6	240	45	95.101
4	0	0	1	-1	0.34375	6	240	30	89.741
5	0	-1	0	1	0.34375	5	145	60	94.611
6	0	1	0	1	0.34375	7	145	60	89.016
7	-1	1	0	0	0.125	7	145	45	57.409
8	0	1	0	-1	0.34375	7	145	30	86.84
9	0	1	-1	0	0.34375	7	50	45	86.736
10	1	-1	0	0	0.5625	5	145	45	96.061
11	0	-1	1	0	0.34375	5	240	45	94.508
12	0	0	0	0	0.34375	6	145	45	91.695
13	-1	0	-1	0	0.125	6	50	45	61.14
14	-1	-1	0	0	0.125	5	145	45	71.068
15	0	0	0	0	0.34375	6	145	45	90.486
16	-1	0	1	0	0.125	6	240	45	67.047
17	0	-1	-1	0	0.34375	5	50	45	93.782
18	0	1	1	0	0.34375	7	240	45	88.601
19	0	0	0	0	0.34375	6	145	45	91.804
20	-1	0	0	1	0.125	6	145	60	66.218
21	0	0	-1	-1	0.34375	6	50	30	87.979
22	0	0	1	1	0.34375	6	240	60	91.71
23	0	0	-1	1	0.34375	6	50	60	88.497
24	-1	0	0	-1	0.125	6	145	30	61.658
25	1	1	0	0	0.5625	7	145	45	93.586
26	0	-1	0	-1	0.34375	5	145	30	93.679
27	1	0	0	-1	0.5625	6	145	30	94.986
28	1	0	0	1	0.5625	6	145	60	94.974
29	0	0	0	0	0.34375	6	145	45	91.192

Table S2
Analysis of variance of decolorization rate according to response surface quadratic model.

Source	Statistical analysis				
	Sum of Squares	df	Mean Square	F-value	p-value
Model	3938.08	14	281.29	446.06	< 0.0001*
A	2832.72	1	2832.72	4492.05	< 0.0001*
B	143.67	1	143.67	227.82	< 0.0001*
C	17.21	1	17.21	27.29	0.0001*
D	8.57	1	8.57	13.6	0.0024*
AB	31.27	1	31.27	49.59	< 0.0001*
AC	6.27	1	6.27	9.95	0.007*
AD	5.23	1	5.23	8.29	0.0121*
BC	0.3243	1	0.3243	0.5143	0.4851
BD	0.3869	1	0.3869	0.6135	0.4465
CD	0.5264	1	0.5264	0.8347	0.3764
A^2	838.78	1	838.78	1330.11	< 0.0001*
B^2	0.2201	1	0.2201	0.349	0.5641
C^2	2.93	1	2.93	4.65	0.0489*
D^2	2.07	1	2.07	3.28	0.0918
Residual	8.83	14	0.6306		
Lack of Fit	7.47	10	0.7468	2.19	0.2333
Pure Error	1.36	4	0.3402		
Cor Total	3946.91	28			

Table S3
Model summary statistics.

Index	Standard deviation	Mean	C.V. %	R^2	Adjusted R^2	Predicted R^2	Adequate Precision
D_r(%)	0.7941	86.04	0.9230	0.9978	0.9955	0.9886	65.9209

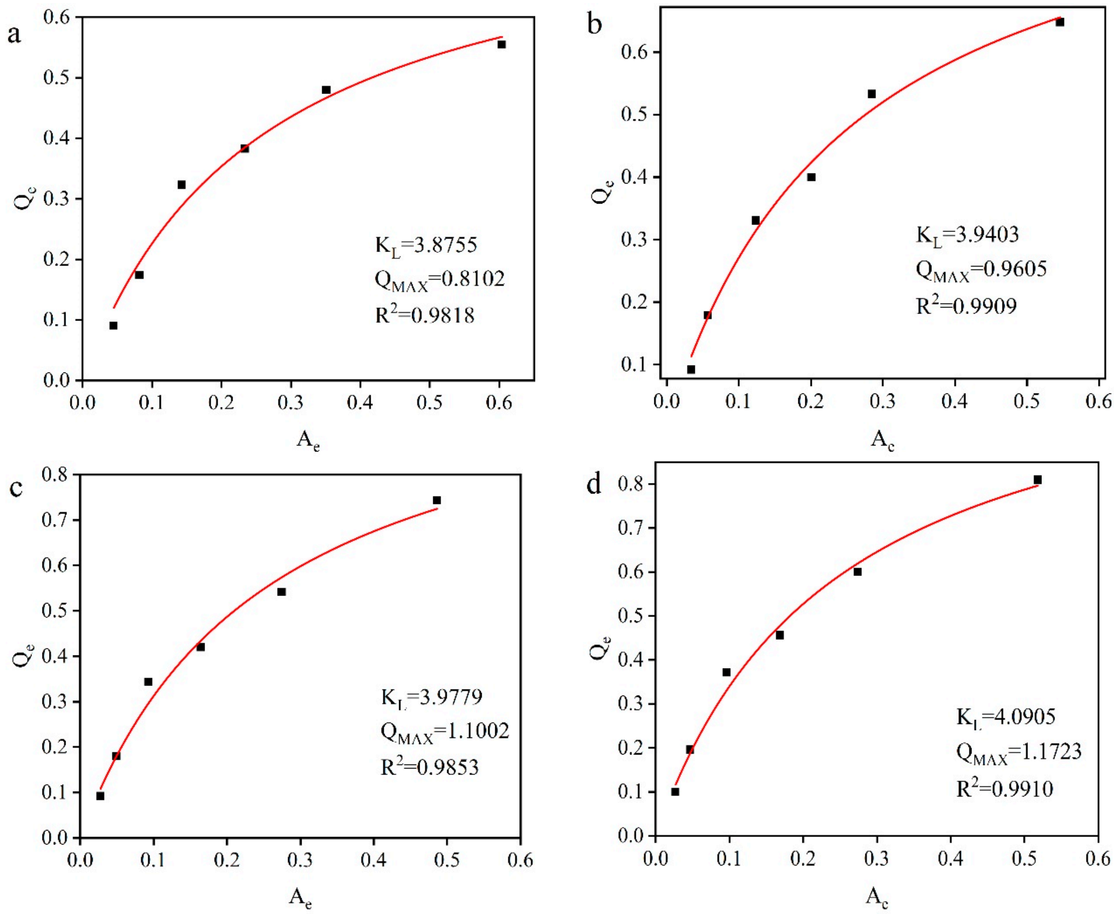

Figure S1. Langmuir model fitting at different temperature conditions, 25°C (a), 40°C (b), 50°C (c), and 70°C (d). The experiments were carried out at pH 5.0 with a decolorization time of 50 min.
